# Influences for Gender Disparity in Academic Psychiatry in the United States

**DOI:** 10.7759/cureus.2514

**Published:** 2018-04-22

**Authors:** Muhammad H Sheikh, Amna Mohyud Din Chaudhary, Anum S Khan, Muhammad A Tahir, Hafiz A Yahya, Sadiq Naveed, Faisal Khosa

**Affiliations:** 1 Psychiatry, King Edward Medical University, Lahore, PAK; 2 Psychiatry, Nishtar Medical College & Hospital, Multan, PAK; 3 Psychiatry, Allama Iqbal Medical College, Lahore, PAK; 4 Psychiatry, SUNY Upstate Medical University, Syracuse, USA; 5 Psychiatry, KVC Hospitals, Kansas City, USA; 6 Department of Radiology, Vancouver General Hospital, Vancouver, CAN

**Keywords:** research, academic, psychiatry, women, usa

## Abstract

Introduction

Academic undertakings, including research, lead to career progression. However, the career paths of female psychiatrists appear to diverge significantly from that of their male counterparts. This article reviews the pervasiveness of the trend of women being less likely to pursue active research in psychiatry. In addition, we examine the correlation between academic rank and research productivity.

Methods

We searched the American Medical Association’s (AMA) Fellowship and Residency Electronic Interactive Database (FREIDA) to identify training programs for psychiatry. A total of 5234 psychiatrists met our inclusion criteria. The gender, academic rank, research work, and h-index of faculty members were compared. The ratio of women reaching senior ranks as compared to men was also calculated. The Scopus database was used to determine the h-index of the individuals included in this study. Data analysis was done with SPSS 22.0 Release 2013 (IBM SPSS Statistics for Windows, IBM, Armonk, NY, USA). Kruskal–Wallis and Mann–Whitney U tests were used where required, with the P-value set at less than 0.05.

Results

In our study sample, 2181 (42%) of the psychiatrists were women. However, according to the information obtained from the websites of 23 programs, few women reached higher ranks, full professorship, or positions such as the chairperson of a program, and only 9% of women achieved the designation of chairperson of the psychiatry department, with men representing the other 91%. Higher academic rank correlated with higher h-index. A statistically-significant difference between the genders in terms of h-index was found for the assistant professor rank as well. However, this difference was not observed at the level of an associate professor.

Conclusions

Despite adequate representation of women in the academic workforce in psychiatry, there appears to be a discrepancy in the research productivity of the two genders. This study highlights the need for targeted interventions to address gender disparities in academic psychiatry.

## Introduction

Although the practice of psychiatry is not experiencing a shortage of women in the workforce, their presence in senior faculty or leadership positions lags behind that of their male colleagues [1]. There are gender differences in academic faculty ranks, with women being substantially less likely than men to become full professors. Numerous reasons have been suggested for this gender disparity in career advancement. Women’s professional activities differed from that of men’s: practice patterns among the former may not be the same, with fewer women opting to join academic psychiatry, and they may have fewer publications in peer-reviewed journals [2]. Gender differences in career paths in psychiatry are affected not only by individual traits and choices, but also by economic factors.

There are a number of reasons why fewer female psychiatrists pursue research careers compared to their male counterparts [2]. One of the possible reasons is the time demands of sustaining a research track, despite the fact that academic pursuits do offer some relief from the constraints of full-time clinical work. Roeske found that female psychiatrists who interrupted their training to have children were more likely to work 30% fewer hours than men [3]. Those who worked part-time or took an extended leave may also be at a disadvantage in academia, where publications and continuous research funding are key factors to career development [1].

The lack of female role models may be a confounding factor [1]. Because society comparatively undermines women’s careers, their need for mentors to help identify strengths and resources may actually be greater than men’s. Non-mentored or under-mentored faculty are less likely to think strategically about their career development, and this in turn affects their future pursuits [4]. Additionally, female psychiatry residents are less likely to pursue research tracks than men because there are fewer women role models or mentors to inspire them in this direction. In 2002, Bickel et al. reported that only a few medical schools had women leaders who could serve as inspiring role models for female physicians. Their report assessed the implementation of strategies to improve women’s representation in academic positions over four years, and found that the goals were grossly underachieved, highlighting the dire need for women role models for aspiring physicians [5].

In this paper, we aim to investigate the current proportion of female psychiatrists in academic faculty positions across the US, and to review the role of female psychiatrists in academic psychiatry. We also provide insights into gender disparities in research contributions to the field of psychiatry.

## Materials and methods

We searched the American Medical Association’s (AMA) Fellowship and Residency Electronic Interactive Database (FREIDA) for psychiatry training programs in the US. Faculty listings from the online sites of these programs were used to obtain academic ranks. Additionally, faculty members were classified by gender, as agreed upon by all authors based on names and pictures from online faculty listings.

Non-academic clinicians, instructors, and voluntary, adjunct, part-time, and non-physician faculty members were excluded from this analysis. Faculty members whose academic rank was not available through online faculty listings and departmental websites were also excluded. After the application of the exclusion criteria, 5234 academic psychiatrists were included from 211 programs identified in the initial FREIDA search.

We used the Scopus biomedical database to collect data about research productivity as measured by the number of articles published, the number of citations, and the h-index. Scopus was selected as a tool to determine the h-index because it was found to offer consistently more coverage than Web of Science and greater accuracy than Google Scholar [6]. Data collection was completed in June 2017. The data were analyzed with IBM SPSS Statistics Version 22.0 Release 2013 (IBM SPSS Statistics for Windows, IBM, Armonk, NY, USA). Kruskal–Wallis and Mann–Whitney U-tests were used for statistical analyses where appropriate, with significance thresholds set at P < 0.05.

## Results

**Figure 1:**
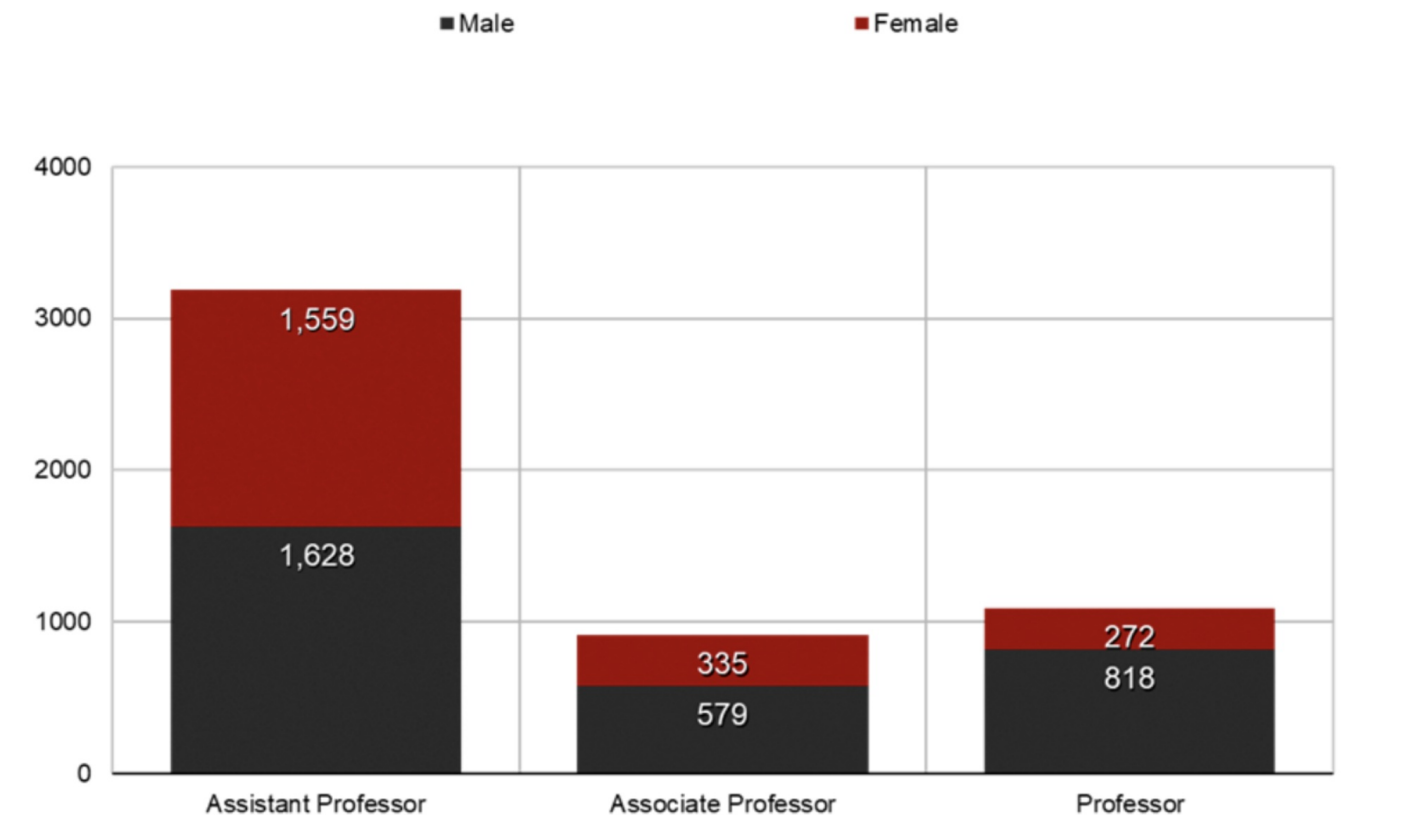
Gender distribution by academic ranks.

Our data analysis of 5234 academic psychiatrists comprised 2181 (42%) women and 3053 (58%) men. Gender distribution by academic ranks is shown in Figure 1. It can be seen that women were less-represented among senior academic ranks. Twenty-three of the programs included in this analysis openly disclosed details on the position of chair or chief of their program; this information indicated that most chairpersons (91%) were men. On the other hand, a majority of the program directors (65%) were women. 

**Figure 2:**
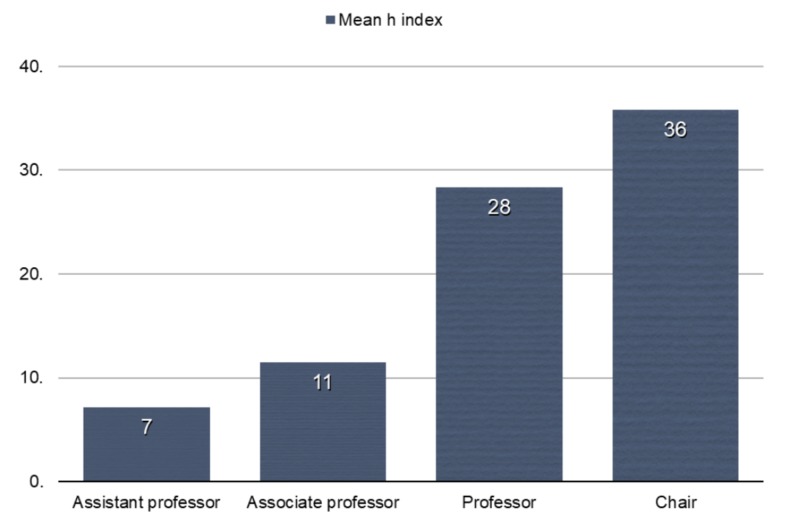
H-index for successive academic ranks.

Male and female research productivity in our study was compared according to h-indexes. Male psychiatrists had a significantly higher overall mean h-index, as shown in Figure 2 (Mann–Whitney U-test, P < 0.01). 

**Figure 3:**
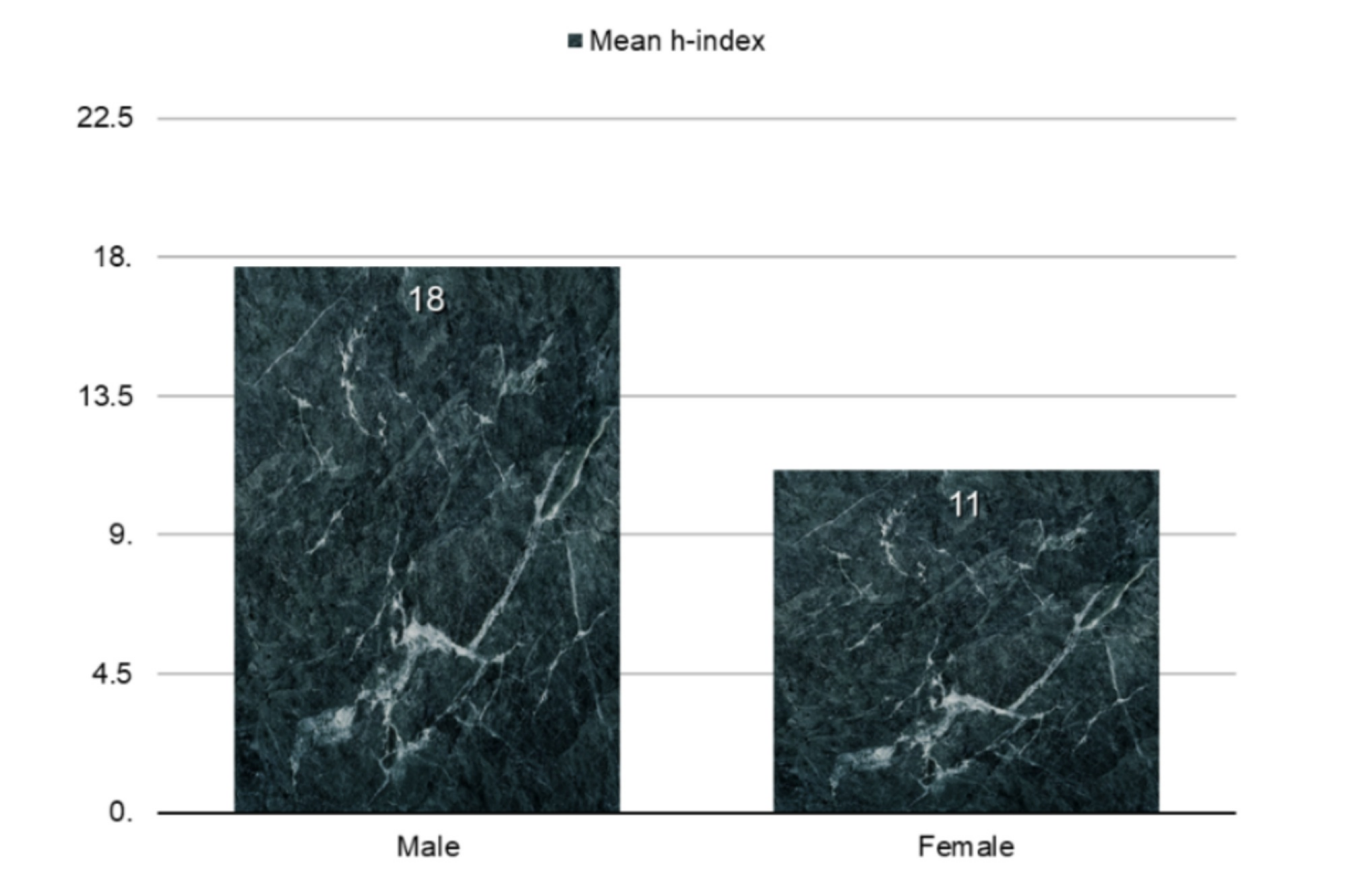
Male and female research productivity.

The h-index of academic psychiatrists increased with successive academic ranks from assistant professor through full professor (Kruskal–Wallis test, P < 0.01) (Figure 3). There was no significant difference in h-index between professors and department chairs (Mann–Whitney U-test, P = 0.41) (Figure 3).

**Figure 4:**
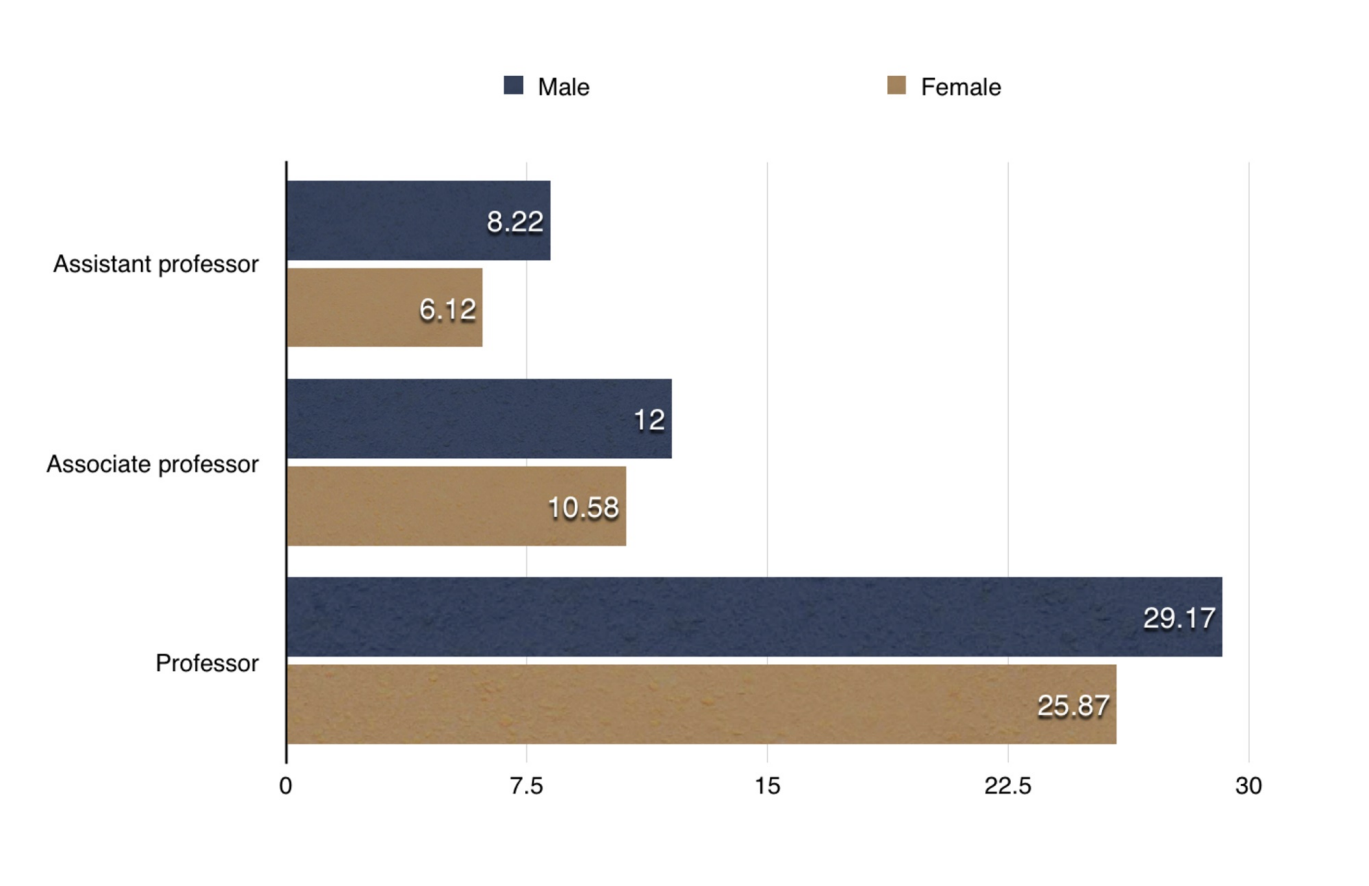
Gender-wise comparison of h-index for different academic ranks.

Upon further examination on the basis of gender, the h-indexes differed significantly between genders at the level of assistant professor (Mann–Whitney U-test, P = 0.012) (Figure 4), whereas no statistically-significant difference was found at the level of associate professor (Mann–Whitney U-test, P = 0.631) (Figure 4) or full professor (Mann–Whitney U-test, P = 0.279) (Figure 4).

## Discussion

According to our data, women comprise 42% of psychiatry faculty members, which is an improvement from only 37% in 2002 [3]. Despite this overall increase, women in psychiatry remain grossly under-represented in the ranks of associate professor and full professor, as well as in leadership positions such as department chairs and deans. Although a significant percentage of women become assistant professors, the proportion of women who subsequently attain higher academic ranks showed a sharp decline (Figure 1). A literature review showed that the number of women reaching the rank of full professor in medical schools has not increased since 1980 [7]. In 1991, the percentage of men who had reached the rank of professor (22%) was more than twice the percentage of women (10%). Only eight women had chaired a medical school psychiatry department, and no female psychiatrists had been dean of a medical school in the USA [3].

Disparities at different levels of the academic hierarchy may have different origins and drivers. For example, lower rates of promotion to associate professorship among women may stem from differences in the choice of career track [7]. Female psychiatrists more often enter the clinical track, which has a slower rate of promotion compared to the research track [7]. Another possible reason for this predilection for clinical career routes may be the greater demand for female psychiatrists in areas with larger immigrant populations, whose members may prefer to talk to same-sex psychiatrists. However, this theory has not been corroborated by research. In summary, differential household responsibilities [8] and different preferences for work–life balance are important contributors to gender differences in full professorship rates [9].

Female faculty usually have lower average research productivity compared to men, which adversely affects their career progression [10]. Our comparison of male and female research productivity disclosed a statistically significant difference (P = 0.012) at the level of assistant professor. A previous survey of 1923 full-time psychiatry department faculty members by Leibenluft and colleagues concluded that nearly three-quarters of male psychiatrists in academic settings had conducted research, compared to about half of female psychiatrists [11]. Furthermore, Leibenluft and colleagues found that men were twice as likely as women to be principal investigators on peer-reviewed grants, or to have mentored research trainees. Despite some progress over the following years, female researchers in psychiatry still fall well behind their male counterparts. In 2001, there were 574 male psychiatrists who were principal investigators on grants, compared to only 122 female investigators (i.e., 18% of psychiatrist-investigators were female) [12].

A study by Kaatz and colleagues also suggested that grant panels might show bias against female applicants [13]. Unconscious bias by both men and women has long been suspected to be an important factor in the review system. Another study by Waisbren et al. evaluated gender differences in the acquisition of research grants by male and female faculty at eight Harvard Medical School-affiliated institutions. This study included 6319 grant proposals from 2460 investigators, among which women comprised 29% of the applicants and submitted 26% of the grant applications [14]. These authors concluded that gender disparity in grant funding correlated with gender disparities in academic rank. When rank was controlled for, women and men were equally successful in acquiring grants, although the submission rates of women were significantly lower at the lowest faculty rank. Although there was no difference in the proportion of money awarded to the money requested, women were awarded significantly less money than men at the ranks of instructor and associate professor. Also, more men than women applied for funding from the National Institutes of Health [14].

Apparently, women often have to make decisions about their family commitments during the same years when their commitment to research is expected to be strongest. This makes the pursuit of an academic and research career challenging. Women also face difficulties in finding effective mentors and receiving recognition from senior colleagues [1,15-17]. At the same time, women may face workplace discrimination and inequitable allocation of institutional resources [18-19]. These challenges adversely affect research productivity and may also explain why even after adjusting for research productivity, women are still less likely than men to be full professors [20].

The main limitation of our study was the possibility of missing data. We relied upon data available from in FRIEDA and Scopus. The number of psychiatry facilities listed in FREIDA was noted to vary by ±5 during the period of data collection for our study. Scopus covers approximately 15,000 peer-reviewed journals and is considered to be more reliable than other sources [6]. Nevertheless, the possibility that some publications in lesser-known journals were inadvertently excluded cannot be ruled out. Also, there is a possibility that missing data might have introduced systematic bias, affecting our results.

## Conclusions

Women continually face barriers to their advancement through academic and professional ranks. A number of explanations have been proposed to explain the gender disparities in faculty rank positions. Interventions are needed to address these barriers, requiring an increase in investments in early research career development for women, and a modification of the promotions process in non-research career tracks. Further studies are also needed to establish the underlying causes of career differences between women and men in psychiatric practice, so that effective strategies can be implemented to correct the current inequalities affecting women in senior faculty and research positions.
